# The AI Will See You Now: Feasibility and Acceptability of a Conversational AI Medical Interviewing System

**DOI:** 10.2196/37028

**Published:** 2022-06-27

**Authors:** Grace Hong, Margaret Smith, Steven Lin

**Affiliations:** 1 Stanford Healthcare AI Applied Research Team Division of Primary Care and Population Health Stanford University School of Medicine Redwood City, CA United States

**Keywords:** artificial intelligence, feasibility studies, patient acceptance of health care, diagnostic errors, patient-generated health data, clinical, medical history, healthcare, health care

## Abstract

**Background:**

Primary care physicians (PCPs) are often limited in their ability to collect detailed medical histories from patients, which can lead to errors or delays in diagnosis. Recent advances in artificial intelligence (AI) show promise in augmenting current human-driven methods of collecting personal and family histories; however, such tools are largely unproven.

**Objective:**

The main aim of this pilot study was to evaluate the feasibility and acceptability of a conversational AI medical interviewing system among patients.

**Methods:**

The study was conducted among adult patients empaneled at a family medicine clinic within a large academic medical center in Northern California. Participants were asked to test an AI medical interviewing system, which uses a conversational avatar and chatbot to capture medical histories and identify patients with risk factors. After completing an interview with the AI system, participants completed a web-based survey inquiring about the performance of the system, the ease of using the system, and attitudes toward the system. Responses on a 7-point Likert scale were collected and evaluated using descriptive statistics.

**Results:**

A total of 20 patients with a mean age of 50 years completed an interview with the AI system, including 12 females (60%) and 8 males (40%); 11 were White (55%), 8 were Asian (40%), and 1 was Black (5%), and 19 had at least a bachelor’s degree (95%). Most participants agreed that using the system to collect histories could help their PCPs have a better understanding of their health (16/20, 80%) and help them stay healthy through identification of their health risks (14/20, 70%). Those who reported that the system was clear and understandable, and that they were able to learn it quickly, tended to be younger; those who reported that the tool could motivate them to share more comprehensive histories with their PCPs tended to be older.

**Conclusions:**

In this feasibility and acceptability pilot of a conversational AI medical interviewing system, the majority of patients believed that it could help clinicians better understand their health and identify health risks; however, patients were split on the effort required to use the system, and whether AI should be used for medical interviewing. Our findings suggest areas for further research, such as understanding the user interface factors that influence ease of use and adoption, and the reasons behind patients’ attitudes toward AI-assisted history-taking.

## Introduction

Primary care providers (PCPs) face numerous challenges, including time constraints and burnout, and are often limited in their ability to collect detailed medical histories from their patients [1]. Currently, patients’ personal and family medical histories are collected via paper forms or interviewing prior to and/or during a patient visit, with manual data entry into the electronic health record (EHR). Often, data are missing or of poor quality due to lack of time or lack of training [2].

Information gaps can lead to errors or delays in diagnosis and failures to address actionable risk factors, which affect an estimated 12 million Americans each year [3]. Diagnosis in primary care is a high-risk area for errors, for several reasons. PCPs typically face high patient volumes, make decisions amid uncertainty, and must balance the risk of missed or delayed diagnoses with the stewardship of scarce resources [4]. Poor communication leading to gaps in information sharing is a major driver of diagnostic errors in primary care settings [4], while engaging and empowering patients in the task of generating data using technology aims to close those gaps and support personalized medicine [5].

Recent advances in conversational agents powered by artificial intelligence (AI) and natural language processing show promise in augmenting current human-driven methods of collecting personal and family histories as part of pre-visit planning [6]. Various types of conversational agents have emerged, including chatbots, embodied conversational agents (avatars), and voice assistants, all of which mimic human conversation using text and/or spoken language [7]. Within health care, these agents have been used for facilitating screening for health conditions, triage, counseling, self-management of chronic conditions, and training for health care professionals; reported benefits have included their potential to support populations with poor health literacy, be scaled to reach large populations, and improve patient engagement [8]. However, such tools remain largely unproven in real-world settings, and few studies have assessed the use case of collecting patients’ information before an appointment to provide tailored counseling [2,9-11]. This study aimed to assess the feasibility and acceptability of a conversational AI medical interviewing system from the perspective of primary care patients.

## Methods

### Setting and Participants

The study was conducted between February and April 2021 at a family medicine clinic within a large academic medical center in Northern California. Participants were eligible to participate if they were aged ≥18 years and English-speaking. Research staff contacted eligible participants via email and telephone, providing a brief summary of the study and asking whether they would be interested in participating.

### Ethical Considerations

The Stanford University Institutional Review Board reviewed this study and exempted it (protocol number IRB-59555).

### Procedure

**Figure 1:**
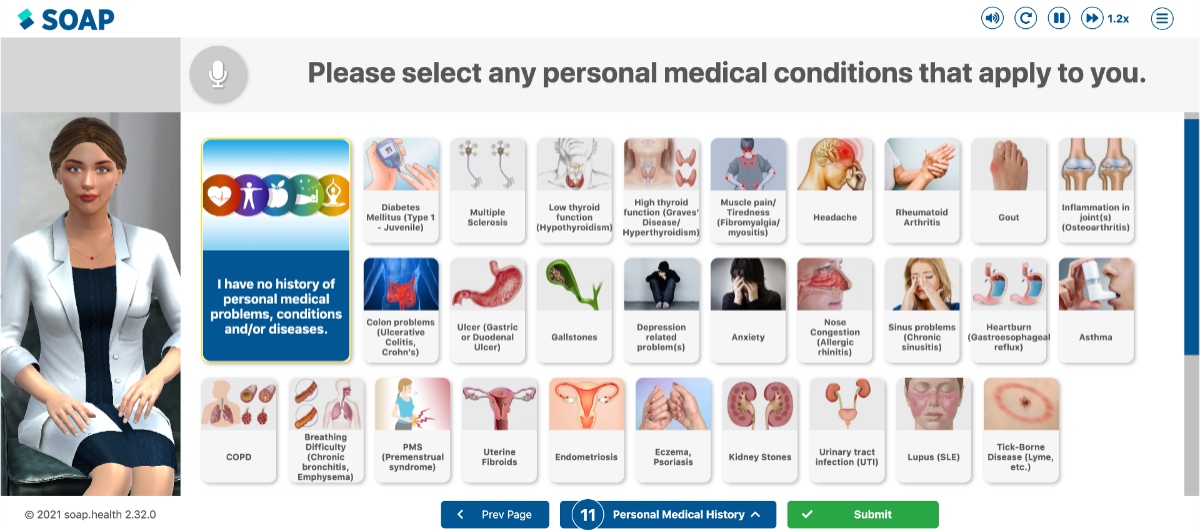
Screenshot of Genie and the conversational AI medical interviewing system. AI: artificial intelligence.

Participants were asked to test the web-based AI medical interviewing system on a personal computer at a time and location of their choosing. The program developed by SOAP Health uses AI and natural language processing to convert speech to text and provide appropriate responses to user-entered data through Genie, a user-facing conversational avatar and chatbot. Genie asked participants a series of questions to (1) capture detailed personal medical histories, multigenerational family histories, and social determinants of health data (eg, financial insecurity, food insecurity, access to affordable health care, access to transportation); and (2) identify risk factors based on established guidelines for further evaluation (eg, hereditary cancers, cardiovascular disease), based on personal or family histories (Figure 1 and Multimedia Appendix 1). Questions covered 25 topic areas and were both asked aloud by Genie and displayed visually onscreen (Multimedia Appendix 1). Participants were able to respond by speaking or clicking a displayed response option and were allowed to use as much time as needed for the interview; most completed it in 30-45 minutes.

### Survey and Outcomes

After completing an interview with the AI system, participants were asked to complete a web-based survey based on the validated Unified Theory of Acceptance and Use of Technology framework [12], a model explaining user acceptance and adoption of new technology that has been used across various studies assessing emerging technology [13-15]. The outcomes were patient-reported feasibility and acceptability ratings from the survey based on responses to Likert-scale questions in the following domains: (1) performance expectancy, the degree to which patients believe using the system will help them share relevant information with their PCPs and identify disease risks earlier; (2) effort expectancy, the degree of ease patients associate with using the system; and (3) attitude toward using technology, the degree to which patients have a positive attitude toward using the system. Participants provided responses on a 7-point scale (strongly disagree to strongly agree) to statements including “Using the software could contribute to my doctor having a better overall understanding of my health and health risks,” “I was able to quickly learn how to use the software,” and “Using artificial intelligence to conduct medical interviewing is a good idea.” The survey also collected patients’ demographic information including their age, gender, race/ethnicity, and highest level of education. Descriptive statistics were used to compute counts with percentages for survey responses.

## Results

Twenty patients with a mean age of 50 years completed an interview with the AI system, including 12 females (60%) and 8 males (40%); 11 were White (55%), 8 were Asian (40%), 1 was Black (5%), and 19 had at least a bachelor’s degree (95%) (Multimedia Appendix 1).

**Figure 2:**
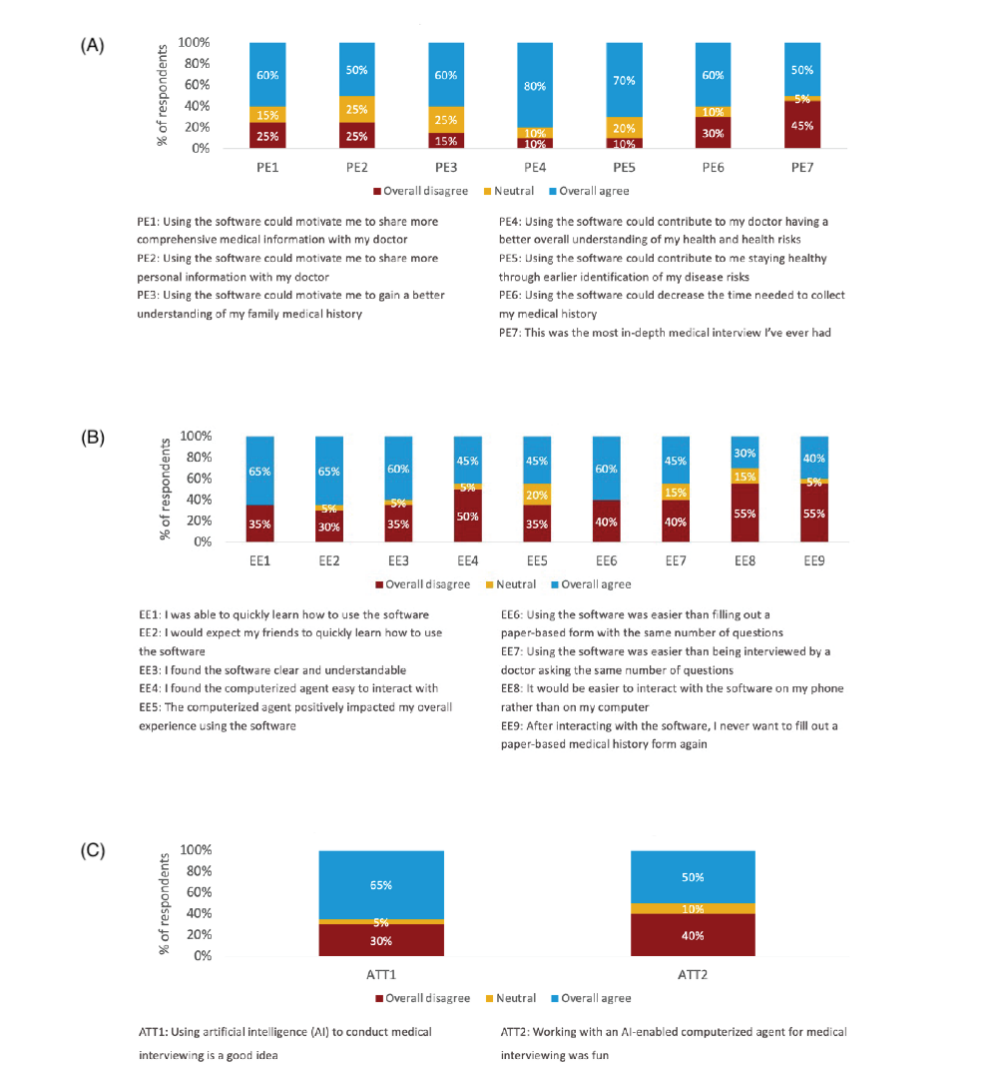
Patient-reported feasibility and acceptability ratings of the system. AI: artificial intelligence; PE: performance expectancy; EE: effort expectancy; ATT: attitude toward using technology.

The majority of participants agreed that using a conversational AI medical interviewing system to collect histories could help PCPs have a better understanding of their health (16/20, 80%) and help them stay healthy through identification of their health risks (14/20, 70%) (Figure 2). Participants who felt the system was easy to use tended to be younger. The median age for those who agreed that they were able to learn the system quickly was 41 years compared to 61 years for those who disagreed and 41 years for those who agreed that the system was clear and understandable compared to 68 years for those who disagreed (Multimedia Appendix 1). Those who reported that the tool could motivate them to share more comprehensive medical information with their PCPs tended to be older—median age of 52 years for those who agreed and 36 years for those who disagreed (Multimedia Appendix 1). Patients were split on the effort required to use the tool, and on whether AI should be used for medical interviewing (Figure 2).

## Discussion

In this feasibility and acceptability pilot of a conversational AI medical interviewing system, the majority of primary care patients believed that such a tool could help PCPs better understand their health and identify their health risks. However, while performance expectancy of the system was predominantly positive, results were mixed in terms of effort expectancy and attitude toward the emerging technology.

Our results are aligned with existing literature. A recent systematic review examining the effectiveness and usability of AI-based conversational agents in health care found that 67% of the 31 studies reported positive or mixed evidence supporting the effectiveness, usability, and positive user perceptions of the agents [8]. Additionally, in a study exploring the acceptability and feasibility of a virtual counselor to collect family health histories in an underserved population, a vast majority of participants found the virtual counselor easy to use and understood the questions being asked [2]. At the same time, studies reporting qualitative feedback have consistently cited the following as barriers that will need to be addressed before conversational agents can be deployed and used widely: agents having difficulty understanding users, agents being repetitive and not sufficiently interactive, and users having difficulty forming connections with the agent [8]. Our study adds to this growing body of evidence by assessing the use of conversational agents in a slightly older population in primary care.

Our study has several limitations including the small sample size and convenience sampling recruitment approach. Those who opted to participate may have had more positive notions about new technologies and may have evaluated the system more favorably than the general population. Moreover, participants in our study were highly educated, which may limit the generalizability of our findings.

The use of conversational agents to gather more detailed information about patients’ personal medical history, family medical history, and social determinants of health could aid in capturing a more holistic view of patients and identifying disease risks earlier. Our findings suggest areas for further research, such as understanding the user interface factors that influence ease of use and adoption, and the reasons behind patients’ dichotomous attitudes toward AI-assisted history-taking.

## Appendix

Multimedia Appendix 1 Additional information about the artificial intelligence medical interviewing system; demographics of participants; and additional information on patient-reported feasibility and acceptability ratings of the system.

## Abbreviations

AI: artificial intelligence
PCP: primary care provider

## Prior Presentations

This project was presented at the North American Primary Care Research Group (NAPCRG) Annual Virtual Meeting (November 19-23, 2021) and the Society of Teachers of Family Medicine (STFM) Annual Spring Conference (April 30-May 4, 2022).
