# Supplementary material for: The AI Will See You Now: Feasibility and Acceptability of a Conversational AI Medical Interviewing System
Source: JMIR Form Res. 2022 Jun 27;6(6):e37028. doi: 10.2196/37028 (PMC9274383; doi:10.2196/37028)
Supplement: Multimedia Appendix 1 [file formative_v6i6e37028_app1.docx]

APPENDIX

Additional information about the AI medical interviewing system

Developed by SOAP Health (Boca Raton, FL), the system uses a voice-based, AI-powered conversational avatar and chatbot to: (1) capture detailed personal medical histories, multi-generational family histories, mental health, and social determinants of health data; and (2) identify patients with risk factors who meet established guidelines for further evaluation (e.g., hereditary cancers, cardiovascular disease), based on personal or family histories.

The system identifies risk factors based on existing risk guidelines from established organizations such as the US Preventive Services Task Force, American College of Medical Genetics, Family Health Foundation, American Diabetes Association, and the American Heart Association for the following conditions: every known cancer, cancer syndrome, tumor, polyp, arrhythmia, cardiomyopathy, aortography, hypercholesteremia, hyperlipidemia, hypertension, congenital anomaly, SIDS, sudden death, type 2 diabetes, stroke, obesity, osteoporosis, sexually transmitted diseases, and viruses.

Demographics of participants


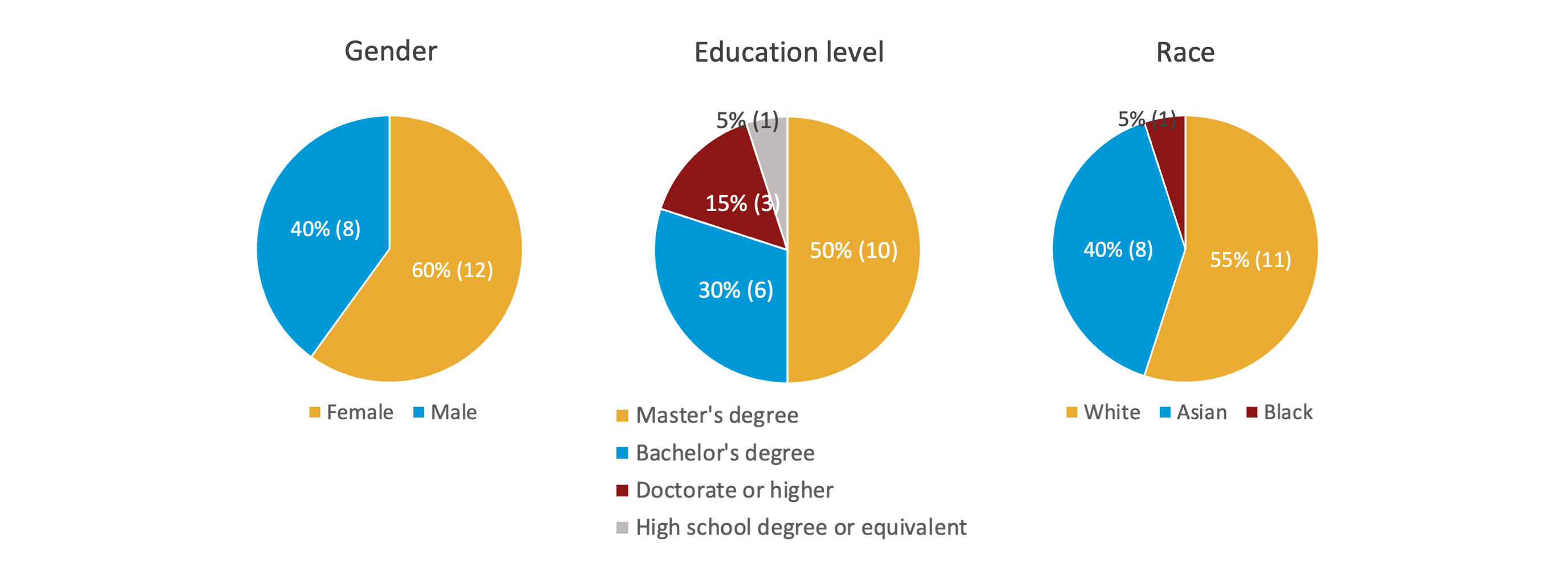


Additional information on patient-reported feasibility and acceptability ratings of the system

Topic areas covered by the AI medical interviewing system

1. Age
2. Gender at birth
3. Gender identity
4. Reason for visit
5. Cardiac symptoms
6. Medications
7. Personal cancer history
8. Personal heart disease history
9. Personal genetic testing
10. Personal medical history
11. Personal surgical history
12. Allergies
13. Social history
14. Adoption
15. Ethnicities and ancestries
16. Family history of cancer
17. Family cancer genetics
18. Family history of heart diseases
19. Family heart genetics
20. Other genetic disorders
21. Major health problems
22. Children of blood related parents
23. Twins/triplets in the family
24. Hereditary concerns
25. Family consult
